# Knowledge of the Population about Visceral Leishmaniasis Transmission in Endemic Areas near the Banks of the Mossoró River in Northeastern Brazil

**DOI:** 10.3390/ijerph120303343

**Published:** 2015-03-19

**Authors:** Camila Fernandes de Amorim, Sthenia Santos Albano Amóra, Thaís Aparecida Kazimoto, Kalídia Felipe de Lima Costa, Luanna Fernandes Silva, Maressa Laíse Reginaldo de Sousa, Yannara Barbosa Nogueira Freitas, Nilza Dutra Alves, Francisco Marlon Carneiro Feijó

**Affiliations:** Programa de Pós-Graduação em Ambiente, Tecnologia e Sociedade—Universidade Federal Rural do Semi-Árido, Rua José Acrísio, 37, Abolição 1, 59619-250 Mossoró, Rio Grande do Norte, Brazil; E-Mails: sthenia@ufersa.edu.br (S.S.A.A.); thaiskazimoto@hotmail.com (T.A.K.); kalidialima@hotmail.com (K.F.L.C.); luannaf@hotmail.com (L.F.S.); maressalaise@gmail.com (M.L.R.S.); yannara_freitas@hotmail.com (Y.B.N.F.); nilzadutra@yahoo.com.br (N.D.A.); marlon@ufersa.edu.br (F.M.C.F.)

**Keywords:** kala-azar, longipalpis, environmental factors, population knowledge

## Abstract

Visceral leishmaniasis (VL) is an anthropozoonosis with high prevalence and incidence in the Northeastern region of Brazil. This study aimed to determine whether people living near the Mossoró River in the city of Mossoró, Rio Grande do Norte, have knowledge of VL and to characterize the environmental properties of this region. Questionnaires were administered to 478 residents in three neighborhoods near the Mossoró River, addressing the population’s knowledge about VL and environmental characteristics. Most survey participants were female, with ages between 18 and 40 years, 53.8% had completed primary education, and 61.5% owned pet dogs (*p* < 0.05). The majority (95.9%) showed little knowledge about the characteristics inherent to sandflies, and 85.3% were unaware of the environments preferred by this vector (*p* < 0.05). Sewage from the homes of respondents was mainly dumped into the river (44.6%), and 76.6% of the respondents complained about the accumulation of garbage in the streets (*p* < 0.05). The association between education and knowledge about the transmission of VL and preferred vector locations was statistically significant (*p* < 0.05). The lack of knowledge in the population about VL and the residential environment indicates a risk of maintaining sand fly habitats and hence disease transmission.

## 1. Introduction

Visceral leishmaniasis (VL), popularly known as kala-azar, is an anthropozoonosis arising in endemic or epidemic form in the populations of peri-urban or rural areas that have a strong tendency to urbanize [1]. In Brazil, the disease is caused by the protozoan *Leishmania chagasi*, transmitted by sandflies of the genus *Lutzomyia* [2,3]. The Northeast region of Brazil has a high incidence of VL, with 47% of reported human cases in both urban and rural areas [4]. The state of Rio Grande do Norte has experienced an increase in the area affected by the disease, demonstrated by the increase in the number of municipalities reporting cases in recent years [5]. The WHO describes VL as an important public health problem in the state of Rio Grande do Norte, with a high incidence rate, high mortality rate and widespread distribution in the state [6]. Other results suggest that the state of Rio Grande do Norte is an important endemic area for VL [7,8].

The increasing number of cases of VL has been associated with the process of urbanization, overcrowded cities, poverty and inadequate sanitation [9,10,11]. The transmission dynamics of VL are associated with a variety of complex eco-epidemiological settings, which offer suitable conditions for interactions between parasite, vector and reservoirs [12]. For this reason, it is important to characterize areas near rivers and ponds to understand the environment and how it supports the maintenance of the VL vector. A study in Iran showed that the high susceptibility areas for VL outbreaks account for 36.3% of the studied area and occurred mainly in the north (which may affect the neighbouring countries) and south (which is a warning for other provinces in Iran), and these parts of the study area had many nomadic, riverside villages [13].

The species of the greatest epidemiological importance in the transmission of VL in the Brazilian territory is the sand fly *Lutzomyia longipalpis* [14,15]. This species easily adapts to urban environments, and its distribution pattern in urban areas can most likely be explained by the degradation of the environment and the presence of humans as a potential source of food [16]. However, other environmental features, such as the presence of domestic animals, fruit trees and unsanitary conditions can also increase the abundance of sand flies, as they provide feeding and breeding places for these insects [17,18].

For these reasons, this study aimed to determine whether the people living in areas near the Mossoró River in the city of Mossoró, Rio Grande do Norte, Brazil, have knowledge of VL. It also sought to understand the environmental characteristics of the study area and to examine whether these characteristics favor the presence of the vector in these areas, supporting the transmission cycle of VL and therefore strengthening the endemicity of the disease.

## 2. Material and Methods

### Study Area

The research took place in the town of Mossoró (37°20'39" W, 05°11'15" S and 16 m above sea level), Rio Grande do Norte, Brazil, between July 2012 to June 2013. The town is located 285 km from the state capital of Natal. It has a land area of 2,110.21 km^2^ and a population of 266,758 inhabitants [19]. The average yearly temperature is approximately 27.4 °C, with a maximum of 36.0 °C and minimum of 21.0 °C. The average annual relative humidity is 70%. The climate is hot and semiarid with low rainfall and two well-defined seasons, the rainy season from February to April (703.7 mm/year) and the dry season.

**Figure 1:**
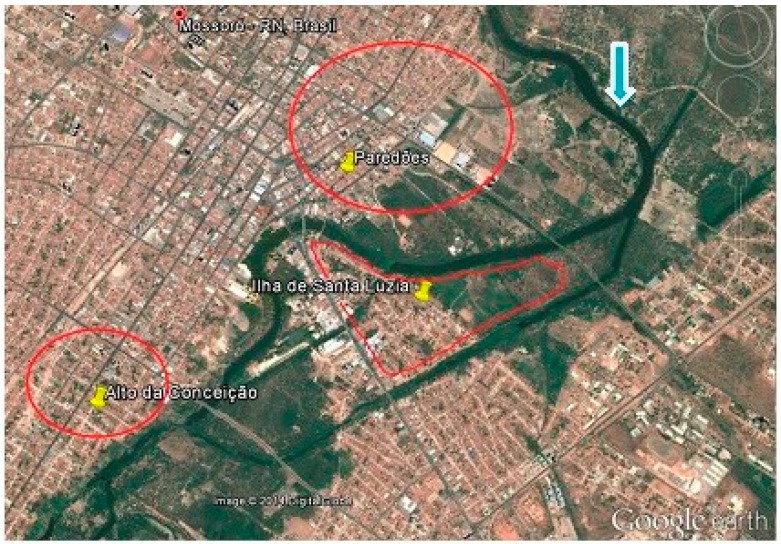
Location of neighborhoods with flood risk (indicated in red) near the Mossoró River (direction of flow indicated by arrow) in the city of Mossoró, State of Rio Grande do Norte. Source: Google Earth—2014, adapted by Amorim, 2014.

The study areas were the neighborhoods at risk of flooding with the elevation of Rio Mossoró in the post-rain period. These data were obtained from the Department of Civil Defense of the Municipality of Mossoró, and are presented in the Damage Assessment Reports of 2008 and 2009. These documents contained, among other information, the report of homes that are near the Rio Mossoró and are at risk of flooding in the rainy season. In these reports, it was observed that the most damage to homes occurred in 2008, resulting from a greater flood than in the last six years. The flood peaked at several neighborhoods in the city, especially the Ilha de Santa Luzia, Alto da Conceição and Paredões neighborhoods (Figure 1). In 2009 the number of neighborhoods in the urban area that suffered damage decreased, concentrating only on the three mentioned neighborhoods. In both floods, 156 homes were hit in Ilha de Santa Luzia neighborhood, in Alto da Conceição 187 and 134 in Paredões. Therefore, this study was conducted in these neighborhoods, and more specifically in homes surrounding the banks of the River Mossoró with identified flood risk, totaling 478 residences, all included in the study.

## 3. Study of the Population’s Knowledge About the Transmission of VL

For each of the 478 residences of the three study districts, an interview was conducted with one resident 18 years or older of either gender who agreed to participate in the study, and who signed an agreement of free and informed consent. The questionnaire included questions about participants’ degree of knowledge about the transmission of VL and items to characterize the participants’ environments. The questionnaire consisted of closed questions with fixed response options, which were separated into categories for analysis. The questions collected demographic data from respondents, with an emphasis on gender, age and education. In addition, information was collected on environmental characteristics, such as the presence of vegetation, garbage and sewage and cleaning methods. The questionnaire also included questions about the presence and care of domestic animals. Finally, the participants were asked about their knowledge regarding the transmission of VL, with questions related to the vector, features of the biology of the insect, its preferred habitats and hours of greatest activity. A questionnaire with open questions was applied as pre-test to validate the questions, with 30 individuals of the same neighborhoods included in the study, being other residents. Based on the responses, the final questionnaire was formulated using as alternative of answers to each question, the answer obtained in the pretest. At the end of each interview, health and environmental education was conducted with the respondent, verbally and individually, based on participant questions and their incorrect responses regarding the disease.

## 4. Data Analysis

Data from participant questionnaire responses were analyzed using the chi-square test (x^2^), using a *p*-value of <0.05 with R software version 3.0.2.

The data were expressed as simple frequencies and percentages calculated using the statistical program SPSS version 21.0 (Statistical Package for Social Science, IBM Corp., Armonk, NY, USA). The association between social variables (gender, age, education) and knowledge of the VL were obtained by Chi-square or Fisher exact test. The latter, in turn, used whenever would arise with values below 5. Values of *p* < 0.05 were expected frequency considered significant.

Statistical significance analyzes the responses of the respondents, and responses were defined as variables. Therefore, the questions were defined as category, and answer for each question were defined as variables. The statistical test was applied separately for each question or category. The answer choices for each question or the variables of each category were compared only among themselves.

## 5. Legal Aspects

The investigation was developed and conducted in compliance with ethical principles with the approval of the State University of Rio Grande do Norte’s Ethics Committee—UERN (CAAE: 03729512.1.0000.5294). All participants signed an informed consent form.

## 6. Results and Discussion

The demographics of the respondents are summarized in Table 1. The majority of participants were female, had an age range of 18–40 years of age and had completed some schooling (*p* < 0.05).

**Table 1:** Details of the social profile of respondents who live in areas near the Mossoró River, in the neighborhoods of Ilha de Santa Luzia, Alto da Conceição and Paredões in the city of Mossoró, Rio Grande do Norte, Brazil, 2013.

Variables Separated by Category ^1^	Number of Responses/Number Total	Percent of Total (%)
**Gender**		
Female	396/478	82.8 *
Male	82/478	17.2
**Age**		
18–40 years	205/478	42.9 *
41–70 years	204/478	42.7
71–100 years	45/478	9.4
No response	24/478	5.0
**Education**		
Completed middle school	257/478	53.8 *
Completed high school	188/478	39.3
Completed college	33/478	6.9

^1^ Variables were separated by category, each category was evaluated separately the Chi-square, and the (*) represents statistical significance (*p* < 0.05).

The social profile of those who answered the questionnaire was comparable to that of similar studies, in which the majority of respondents were female and the mean age was 35 years [20,21]). Most had completed basic schooling, similar to other studies that discussed the association between low educational level and the risk of VL infection [10,20]. Because the present study results corroborate prior research, it is possible to conclude that the low educational level of the population in areas next to the Mossoró River may be susceptible VL infection. The goal was only to characterize the population surveyed, and this analysis was not used for discussion, it was just done a comparison with other similar studies.

Table 2 shows the environmental characteristics of the neighborhoods under study. With respect to vegetation, the vast majority of respondents (*p* < 0.05) stated that there are remnants of forest in the areas around their homes. Respondents also reported the presence of fruit trees in the yards of homes (*p* > 0.05), although this result was not significant. Most reported that garbage was collected by garbage collection trucks (*p* < 0.05), though many also reported the presence of open-air garbage dumps in these areas (*p* < 0.05). The majority of respondents reported that they dumped waste water into the river (*p* < 0.05), and the majority of respondents reported a lack of insecticidal or repellent plants in the area around their homes (*p* < 0.05).

Regarding the presence of mosquitoes in the house, most respondents reported that the region “has a lot of insects”, (86.2%; *p* < 0.05), that in the rainy season the number of insects, including mosquitoes (*Culex* sp.), significantly increases (83.5%; *p* < 0.05) and that insects are most active in the early evening.

Most respondents reported that they lived near areas of vegetation (*p* < 0.05), and it is known that environments with remnants of forest and fruit trees provide suitable breeding sites for sand flies because the insects feed on fallen fruits [17,18]. Thus, the presence of this type of vegetation around the homes of residents is a factor enabling the spread of the vector in these areas. 

**Table 2:** Environmental features around the residences of respondents who live in areas near the Mossoró River, neighborhoods Ilha de Santa Luzia, Alto da Conceição and Paredões in the city of Mossoró, Rio Grande do Norte, 2013.

Variables Separated by Category ^1^	Number of Responses/Number Total	Number Relative (%)
**Lives near areas of vegetation**		
Yes	449/478	93.9 *
No	29/478	6.1
**Destination of Household Waste**		
Garbage truck	288/478	60.3 *
Local dump site	6/478	1.3
Garbage truck and other destinations	184/478	38.5
**Lives near open-air garbage dump**		
Yes	366/478	76.6 *
No	112/478	23.4
**Disposal of domestic sewage**		
Mossoró River	213/478	44.6 *
Sanitation system	181/478	37.9
Sink	70/478	14.6
Sink and Mossoró River	8/478	1.7
Unknown	6/478	1.3
**Vegetation around residence**		
Fruit trees	135/478	28.2
Other types of vegetation	166/478	34.7
Does not have vegetation	177/478	37.0
**Presence of mosquitoes in house**		
Yes	412/478	86.2 *
No	66/478	13.8
**Increase in mosquitoes in the rainy season**		
Yes	399/478	83.5 *
No	79/478	16.5
**Repellent plants/insecticides used near home**		
Yes	15/478	3.1
No	463/478	96.9 *

^1^ Variables were separated by category, each category was evaluated separately the Chi-square, and the (*) represents statistical significance (*p* < 0.05).

The lack of sanitation in the study area is conducive to its maintenance as an environmental vector of VL because the garbage dumps and sewers exposed to the environment provide the type of organic matter that is a food source for immature and adult sand flies [17,18]. The presence of poor housing and environmental sanitation conditions are most likely associated with maintaining the endemicity of VL in these areas, given that the disease tends to affect disadvantaged populations [15]. The areas of study also present an environment rich in sand fly food sources. 

The interior of homes also presents favorable conditions for the presence and reproduction of several species of insects that are active from early evening throughout the night. The improper disposal of solid waste and the presence of spaces that collect standing water in the rainy season are conducive to mosquito breeding. Increased populations of disease-carrying insects, such as sand flies, can eventually cause public health problems [22]. Thus, this population may eventually be exposed to sand flies without knowing the risk to their health.

It is possible that the absence of plants with repellent or insecticidal qualities around homes can be a factor that maintains the presence of insects near the home when associated with the other environmental factors. According to a study, of insecticidal plants, oil from the seeds of *Acalypha indica*, popularly known as neem, has an insecticidal effect on the three stages of development of *L. longipalpis*, and this plant is easily grown in tropical regions [23]. An Ethiopian study of the neem plant concluded that the application of neem oil may be safe for personal use as a low-cost protective measure against sand fly bites for people living in endemic areas [24]. However, a lack of knowledge about the action of these plants most likely prevents them from being used to control the population of VL vectors in specific regions. Encouragement of research in this area may lead to a decrease in the density of the sand fly population.

As shown in Table 3, respondents were asked whether they performed fumigation in their homes, and 163 (34.1%) reported that they used chemicals to prevent insect infestation. The frequency of fumigation was around one to three times per year (*p* > 0.05), and generally, respondents did not know the active ingredient or trade name of the product used (*p* > 0.05). When the population was asked if the fumigation was conducted in homes, was obtained as a response all the possibilities of alternatives included in the questionnaire, including the daily fumigation. However, it was realized that this practice was done randomly and with different types of chemicals, including poisons to kill ants and cockroaches. 

**Table 3:** Information on home fumigation from respondents who live in areas near the Mossoró River, in the neighborhoods of Ilha de Santa Luzia, Alto da Conceição and Paredões, in the city of Mossoró, Rio Grande do Norte, 2013.

Variables Separated by Category ^1^	Number of Responses/Number Total	Percent of Total (%)
**Frequency of fumigation**		
Daily	5/163	3.1
1 to 3× per week	29/163	17.8
1 to 3× per month	61/163	37.4
1 to 3× per a year	68/163	41.7 *
**Chemical products used**		
Pyrethroids	6/163	3.7
Others	76/163	46.6
Unknown	81/163	49.7 *

^1^ Variables were separated by category, each category was evaluated separately the Chi-square, and the (*) represents statistical significance (*p* < 0.05).

This demonstrates the ignorance of the people about the products used specifically to control sand flies such as pyrethroids. This ignorance, therefore, may increase the risk of this population being bitten by females of these vectors. Adult sand fly control measures include the use of spray insecticides in homes and animal shelters [9]. Chemical treatment using insecticides with residual action is a recommended strategy for vector control; however, it only affects the adult insect and aims to prevent and/or reduce the risk of disease transmission [2]. Vector control actions are still the most powerful tools for the prevention of vector-borne diseases [25]. Area residents interviewed for this study were unaware of this information and ultimately did not use this strategy to control the vector of VL, which may cause the persistence of sandflies in the area.

Few respondents reported using synthetic pyrethroids for pest control. These products have been evaluated for the control of VL vectors and are used by health services as a control measure in disease transmission areas [9]. Although residents interviewed for this study used pyrethroids, they do not specifically use these products to control the VL vector because they were unaware of this benefit.

Regarding cleaning the area around the home, 440 (92.1%) of the respondents reported performing this activity (Table 4). It was observed that the cleaning of backyards was performed daily with brooms (*p* < 0.05). Most respondents reported that they did not use chemicals to clean their homes (*p* < 0.05).

**Table 4:** Information on cleaning around the homes of the residences of respondents who live in areas near the Mossoró River, in the neighborhoods of Ilha de Santa Luzia, Alto da Conceição and Paredões in the city of Mossoró, Rio Grande do Norte, 2013.

Variables Separated by Category ^1^	Number of Responses/Number Total	Percent of Total (%)
**Method of cleaning**		
Sweep	389/440	88.4 *
Sweep and wash	50/440	11.4
Weed	1/440	0.2
**Frequency of cleaning**		
Daily	266/440	60.5 *
1 to 3× per week	145/440	33.0
1 to 2× per month	21/440	4.8
2 to 6× per a year	8/440	1.8
**Chemical products used**		
General cleaning products	50/440	11.4
None used	390/440	88.6 *

^1^ Variables were separated by category, each category was evaluated separately the Chi-square, and the (*) represents statistical significance (*p* < 0.05).

We can see that most residents were concerned about maintaining clean yards, though not with the intention of eliminating sand fly habitats. It is known that this is a way to avoid the maintenance of environments that support sand fly populations because, preventive measures for environmental management include the cleaning of backyards, land and public squares to eliminate favorable habitats for the breeding of immature forms of the vector [2].

Respondents were asked whether they raised domestic pets and/or livestock in their backyards, and 265 (55.4%) said yes (*p* < 0.05). *L. longipalpis* is the most common sand fly in the area under study and is found throughout the year in different parts of the city in question [5,26]. These insects are well adapted to environments modified by human activity where chickens and other domestic animals are present, and these factors can increase the abundance of sandflies by providing suitable breeding sites [17].

One finding that merits attention, although it is not statistically significant, is the 68 (25.6%) households that raise chickens. Although chickens are not a confirmed reservoir of Leishmania, chickens are common sources of blood for *L. longipalpis*, and their shelters are known habitats and breeding locations for sand flies, which makes chickens important in the maintenance of vector populations in a given area [27,28]. Thus, the presence of chickens in these areas of study is a hazard to residents.

On the topic of domestic animals in homes, when asked which animals lived with their families in the home environment, 163 (61.5%) respondents reported pet dogs (*p* < 0.05), a fact which confirms the epidemiology of the disease in the country, given that dogs are the main reservoir of *L. chagasi* in urban areas [29,30,31]. In a study conducted in Iran, it was concluded that the low prevalence of VL in humans could be explained by the scarcity of infected domestic dogs [32]. In the study area, residents commonly have contact with dogs, and there is no effective strategy against canine VL infection, which may cause the increased susceptibility of contamination by VL.

Respondents who raised dogs were asked questions about which strategies they used to protect their dogs from sand fly bites. The majority of respondents did not use repellent collars on their dogs (*p* < 0.05). When asked if they walked their dogs at night, most said they did not (*p* > 0.05), although this figure was not statistically significant (Table 5).

**Table 5:** Methods of protecting dogs against sand fly bites in areas near the Mossoró River, in the neighborhoods of Ilha de Santa Luzia, Alto da Conceição and Paredões in the city of Mossoró, Rio Grande do Norte, 2013.

Variables Separated by Category ^1^	Number of Responses/Number Total	Percent of Total (%)
**Use of repellent collars**		
Yes	3/163	1.8
No	160/163	98.2 *
**The dog is walked between 6 pm and 6 am**		
Yes	69/163	42.3
No	94/163	57.7

^1^ Variables were separated by category, each category was evaluated separately the Chi-square, and the (*) represents statistical significance (*p* < 0.05).

The literature states that the use of insecticide-impregnated collars on dogs is one of the measures used to control the population of adult sand flies [9] and that avoiding possible canine infection is a way to maintain distance from the vector. A study carried out in Italy suggests that the mass use of insecticide-impregnated collars led to a reduction of movement of *L. chagasi* in a region [33]. Because few dogs living in the study areas wear collars with repellent action, dogs in the study area may be at risk of infection by VL. Another factor that draws attention is the habit that some have to walk their dogs at night, which is a risk to animals because *L. longipalpis* activity occurs from dusk to dawn [34]. Dogs that are walked when vector activity is high have a higher susceptibility of infection due to the possibility of contact with sandflies in the environment. 

With regard to knowledge about the transmission of disease, 471 (98.5%) respondents said that they had heard of “kala-azar” (*p* < 0.05). Of these, 229 (48.6%) said they did not know the agent of transmission (*p* < 0.05). Residents who responded that mosquitoes transmitted VL (170; 36.1%) were asked other questions about the vector, as shown in Table 6. The association between knowledge of respondents about the transmission of VL and social profile was significant for: the knowledge of “transmitting agent” was associated with age and education of respondents, knowledge of “reported seeing the vector” was associated with the gender of the respondent.

Most respondents knew that mosquitoes transmitted VL, but they reported that they have never seen this insect, did not know its features, were unaware of the environments preferred by the vector and did not know the hours of insect activity (*p* < 0.05), which exposes them to the susceptibility of VL infection. Lack of knowledge on sandfly causes the population to maintain favorable environments for the spread of VL, leaving family and pets exposed to the bite of the vector and the possibility of this sandfly contain the infective forms of the parasite, thus being able to infect people and dogs. Increasing the knowledge of sand fly reproduction habitats may prove useful to vector control efforts, leading to a reduction in the density of vectors and thus decrease disease incidence [35,36].

Research conducted in the state of Belo Horizonte, Brazil, noted the general lack of VL knowledge among the population of the region, indicating an urgent need for the implementation of educational practices [20]. The low knowledge in the study population about the VL vector prevents them from seeking effective control measures for vector-borne disease.

VL researchers in Ethiopia reported that the most important prerequisite for the success of prevention and control of any disease is community participation because the cooperation of the affected population is essential for the implementation and use of program activities [21]. Therefore, it is important that the study population knows about ways to control the VL vector because then they are able to eliminate vector breeding habitats, blocking the chain of transmission. 

The lack of knowledge about basic aspects of VL shows a lack of access to information. A study reports that the preventive measures of VL are impeded during implementation by the absence of a public informed about the basics characteristics of the disease [20]. Services that include measures to control VL should be deployed in health education activities with the population and require the active inclusion of multidisciplinary teams, a practice that so far has not been observed [2]. It is also known that knowledge about sand fly reproduction habitats provides useful information for guiding biological control efforts, leading to a reduction in the density of vectors and thus a reduction of disease incidence [36].

**Table 6:** Respondents knowledge about the vector of visceral leishmaniasis in Mossoró near the river e associação com perfil social, in the neighborhoods of Ilha de Santa Luzia, Alto da Conceição and Paredões in the city of Mossoró, Rio Grande do Norte areas, 2013.

Variables Separated by Category	Total Freq. (%)	Gender Freq. (%)			Age ^a^ Freq. (%)				Education Freq. (%)			
		Female	Male	*p*-Value	18–40 years	41–70 years	71–100 years	*p*-Value ^τ^	Completed Middle School	Completed High School	Completed College	*p*-Value
**Heard about Kala-azar**												
Yes	471 (98.5)	391 (83.0)	80 (17.0)	0.344	203 (44.8)	206 (45.5)	44 (9.7)	0.401	251 (53.3)	187 (39.7)	33 (7.0)	0.226
No	07 (1.5)	05 (71.4)	02 (28.6)		02 (28.6)	04 (57.1)	01 (14.3)		06 (85.7)	01 (14.3)	0 (0.0)	
Total	478 (100)	396 (82.8)	82 (17.2)		205 (44.6)	210 (45.7)	45 (9.8)		257 (53.8)	188 (39.3)	33 (6.9)	
**Transmitting agent**												
Mosquito	170 (36.1)	145 (85.3)	25 (14.7)	0.585	82 (50.9)	65 (40.4)	14 (8.7)	0.046 *	65 (38.2)	87 (51.2)	18 (10.6)	<0.001 *
Incorrectly answered	72 (15.3)	58 (80.6)	14 (19.4)		30 (42.3)	38 (53.5)	03 (4.2)		45 (62.5)	23 (31.9)	04 (5.6)	
Did not know	229 (48.6)	188 (82.1)	41 (17.9)		91 (41.2)	103 (46.6)	27 (12.2)		141 (61.6)	77 (33.6)	11 (4.8)	
Total ^**b**^	471 (100)	391 (83.0)	80 (17.0)		203 (44.8)	206 (45.5)	44 (9.7)		251 (53.3)	187 (39.7)	33 (7.0)	
**Reported seeing the vector**												
Yes	09 (5.3)	05 (55.6)	04 (44.4)	0.028 *	06 (66.7)	03 (33.3)	0 (0.0)	0.494	04 (44.4)	04 (44.4)	01 (11.1)	0.913
No	161 (94.7)	140 (87.0)	21 (13.0)		76 (50.0)	62 (40.8)	14 (9.2)		61 (37.9)	83 (51.6)	17 (10.6)	
Total ^**b**^	170 (100)	145 (85.3)	25 (14.7)		82 (50.9)	65 (40.4)	14 (8.7)		65 (38.2)	87 (51.2)	18 (10.6)	
**Characteristics of the vector**												
Known	07 (4.1)	04 (57.1)	03 (42.9)	0.066	04 (57.1)	03 (42.9)	0 (0.0)	0.704	04 (57.1)	02 (28.6)	01 (14.3)	0.471
Unknown	163 (95.9)	141 (86.5)	22 (13.5)		78 (50.6)	62 (40.3)	14 (9.1)		61 (37.4)	85 (52.1)	17 (10.4)	
Total ^**b**^	170 (100)	145 (85.3)	25 (14.7)		82 (50.9)	65 (40.4)	14 (8.7)		65 (38.2)	87 (51.2)	18 (10.6)	
**Preferred habitats of vector**												
Known	28 (16.5)	25 (89.3)	03 (10.7)	0.770	12 (46.2)	12 (46.2)	02 (7.7)	0.806	06 (21.4)	18 (64.3)	04 (14.3)	0.133
Unknown	142 (83.5)	120 (84.5)	22 (15.5)		70 (51.9)	53 (39.3)	12 (8.9)		59 (41.5)	69 (48.6)	14 (9.9)	
Total ^**b**^	170 (100)	145 (85.3)	25 (14.7)		82 (50.9)	65 (40.4)	14 (8.7)		65 (38.2)	87 (51.2)	18 (10.6)	
**Vector activity times**												
Known	25 (14.7)	24 (96.0)	01 (4.0)	0.131	11 (55.0)	09 (45.0)	0 (0.0)	0.336	08 (32.0)	15 (60.0)	02 (8.0)	0.628
Unknown	145 (85.3)	121 (83.4)	24 (16.6)		71 (50.4)	56 (39.7)	14 (9.9)		57 (39.3)	72 (49.7)	16 (11.0)	
Total ^**b**^	170 (100)	145 (85.3)	25 (14.7)		82 (50.9)	65 (40.4)	14 (8.7)		65 (38.2)	87 (51.2)	18 (10.6)	

**^a^** Number of missing responses; **^b^** Total valid respondents less than *n* = 478; **^τ^** Chi-square test for trend; * Statistical significance (*p* < 0.05).

## 7. Conclusions

In the present study it was observed that the population in areas near the Mossoró River (Rio Grande do Norte, Brazil) has little knowledge about VL and its vector and is unaware of methods to reduce sand fly habitats, which is a health problem to both the people and the dogs living in that environment. Additionally, in these areas, the environment is conducive to the maintenance of VL vector habitats, with many sources of food, abundant organic matter and, given the proximity of the river, favorable moisture levels and temperatures. 

Additionally, low educational attainment may be a factor that influences the knowledge of VL, as it may affect the understanding and implementation of preventive measures against diseases. In the absence of knowledge about the disease, the population tends to maintain favorable environments for the reproduction of the vector, exposing them to the susceptibility of infection. 

Thus, residents of these areas and dogs are constantly exposed to the susceptibility of infection by VL. As a result, health education measures are important for the population, given that these actions can inform and educate the public about the importance of environmental management to eliminate vector breeding sites, especially in environmental contexts that favor the reproduction of the insect.

## References

[1] Marzochi M.C.A., Fagundes A., Andrade M.V., Souza M.B., Madeira M.F., Mouta-Confort E., Schubach A.O., Marzochi K.B.F. (2009). Visceral leishmaniasis in Rio de Janeiro, Brazil: Eco-epidemiological aspects and control. Rev. Soc. Bras. Med. Trop..

[2] Secretaria de Vigilância em Saúde, Ministério da Saúde Manual de Vigilância e Controle da Leishmaniose Visceral. http://portal.saude.gov.br/portal/arquivos/pdf/manual_leish_visceral2006.pdf.

[3] Werneck G.L. (2010). Geographic spread of visceral leishmaniasis in Brazil. Cad. Saúde Pública.

[4] Afonso M.M.S., Duarte R., Miranda J.C., Caranha L., Rangel E.F. (2012). Studies on the feeding habits of *Lutzomyia (Lutzomyia) longipalpis* (Lutz & Neiva, 1912) (Diptera: Psychodidae: Phlebotominae) populations from endemic areas of American Visceral Leishmaniasis in Northeastern Brazil. J. Trop. Med..

[5] Amóra S.S.A., Bevilaqua C.M.L., Dias E.C., Feijó F.M.C., Oliveira P.G.M., Peixoto G.C.X., Alves N.D., Oliveira L.M.B., Macedo I.T.F. (2010). Monitoring of *Lutzomyia longipalpis* Lutz & Neiva, 1912 in an area of intense transmission of visceral leishmaniasis in Rio Grande do Norte, Northeast Brazil. Rev. Bras. Parasitol. Vet..

[6] Barbosa I.R. (2013). Epidemiologia da Leishmaniose Visceral no estado do Rio Grande do Norte, Brasil. Rev. Epidemiol. Control Infect..

[7] Queiroz P.V., Monteiro G.R., Macedo V.P., Rocha M.A., Batista L.M., Queiroz J.W., Jerônimo S.M., Ximenes M.F. (2009). Canine visceral leishmaniasis in urban and rural areas of Northeast Brazil. Res. Vet. Sci..

[8] Barbosa I.R., Costa Í.C.C. (2013). Aspectos clínicos e epidemiológicos da leishmaniose visceral emmenores de 15 anos no estado do Rio Grande do Norte, Brasil. Sci. Med..

[9] Barata R.A., Michalsky E.M., Fujiwara R.T., França-Silva J.C., Rocha M.F., Dias E.S. (2011). Assessment of sand fly (Diptera, Psychodidae) control using cypermethrin in an endemic area for visceral leishmaniasis, Montes Claros, Minas Gerais State, Brazil. Cad. Saúde Pública.

[10] Argaw D., Mulugeta A., Herrero M., Nombela N., Teklu T., Tefera T., Belew Z., Alvar J., Bern C. (2013). Risk factors for visceral leishmaniasis among residents and migrants in Kafta-Humera, Ethiopia. PLOS Negl. Trop. Dis..

[11] Fu Q., Li S.Z., Wu W.P., Hou Y.Y., Zhang S., Feng Y., Zhang L.P., Tang L.H. (2013). Endemic characteristics of infantile visceral leishmaniasis in the People’s Republic of China. Parasit. Vectors.

[12] Vilela M.L., Azevedo C.G., Carvalho B.M., Rangel E.F. (2011). Phlebotomine fauna (Diptera: Psychodidae) and putative vectors of leishmaniases in impacted area by hydroelectric plant, State of Tocantins, Brazil. PLoS One.

[13] Rajabi M., Mansourian U., Pilesjö P., Bazmani U. (2014). Environmental modelling of visceral leishmaniasis by susceptibility-mapping using neural networks: A case study in north-western Iran. Saúde Geospat..

[14] Harhay M.O., Olliaro P.L., Vaillant M., Chappuis F., Lima M.A., Ritmeijer K., Costa C.H., Costa D.L., Rijal S., Sundar S., Balasegaram M. (2011). Who is a typical patient with visceral leishmaniasis? Characterizing the demographic and nutritional profile of patients in Brazil, East Africa, and South Asia. Am. J. Trop. Med. Hyg..

[15] Mccarthy C.B., Santini M.S., Pimenta P.F., Diambra L.A. (2013). First comparative transcriptomic analysis of wild adult male and female *Lutzomyia longipalpis*, vector of visceral leishmaniasis. PLoS One.

[16] Nascimento M.D., Silva M.H., Viana G.M., Leonardo F.S., Bezerra G.F., Silva A.S., Soares V.C., Pereira S.R., Rebêlo J.M., Brasil R.P. (2013). Spatial dynamics of urban populations of *Lutzomyia longipalpis* (Diptera: Psychodidae) in Caxias, State of Maranhão, Brazil. Rev. Soc. Bras. Med. Trop..

[17] Fernández M.S., Salomón O.D., Cavia R., Pérez A.A., Acardi S.A., Guccione J.D. (2010). *Lutzomyia longipalpis* spatial distribution and association with environmental variables in an urban focus of visceral leishmaniasis, Misiones, Argentina. Acta Trop..

[18] Melo S.C., Cella W., Massafera R., Silva N.M., Marqui R., Carvalho M.D.B., Teodoro U. (2013). Phlebotomine sandflies in rural locations in the State of Parana, southern Brazil. Rev. Inst. Med. Trop. São Paulo.

[19] Instituto Brasileiro de Geografia e Estatística—IBGE (2011). Sinopse do Censo Demográfico 2010. Mossoró, Rio Grande do Norte, Brazil. http://www.censo2010.ibge.gov.br/sinopse/index.php?uf=24&dados=0.

[20] Borges B.K., Silva J.A., Haddad J.P., Moreira E.C., Magalhães D.F., Ribeiro L.M., Fiúza V.O. (2008). Assessment of knowledge and preventive attitudes concerning visceral leishmaniasis in Belo Horizonte, Minas Gerais State, Brazil. Cad. Saude Publica.

[21] Alemu A., Alemu A., Esmael N., Dessie Y., Hamdu K., Mathewos B., Birhan W. (2013). Knowledge, attitude and practices related to visceral leishmaniasis among residents in Addis Zemen town, South Gondar, Northwest Ethiopia. BMC Public Health.

[22] Zuluaga W.A., López Y.L., Osorio L., Salazar L.F., González M.C., Ríos C.M., Wolff M.I., Escobar J.P. (2012). Twenty-year surveillance of insects relevant to public health during the construction of hydroelectric facilities in Antioquia, Colombia. Biomedica.

[23] Maciel M.V., Morais S.M., Bevilaqua C.M., Silva R.A., Barros R.S., Sousa R.N., Sousa L.C., Machado L.K., Brito E.S., Souza-Neto M.A. (2010). *In vitro* insecticidal activity of seed neem oil on *Lutzomyia longipalpis* (Diptera: Psychodidae). Rev. Bras. Parasitol. Vet..

[24] Kebede Y., Gebre-Michael T., Balkew M. (2010). Laboratory and field evaluation of neem (*Azadirachta indica A. Juss*) and Chinaberry (*Melia azedarach L.*) oils as repellents against Phlebotomus orientalis and *P. bergeroti* (Diptera: Psychodidae) in Ethiopia. Acta Trop..

[25] Tiwary P., Kumar D., Mishra M., Singh R.P., Rai M., Sundar S. (2013). Seasonal variation in the prevalence of sand flies infected with *Leishmania donovani*. PLoS One.

[26] Amóra S.S.A., Bevilaqua E.C., Feijó F.M.C., Oliveira P.G.M., Peixoto G.C.X., Sousa R.N., Alves N.D., Oliveira L.M.B., Macedo I.T.F. (2010). Sandflies (Psychodidae: Phlebotominae) survey in an urban transmission area of visceral leishmaniasis, Northeastern Brazil. Rev. Bras. Parasitol. Vet..

[27] Alexander B., Carvalho R.L., McCallum H., Pereira M.H. (2002). Role of the domestic chicken (*Gallus gallus*) in the epidemiology of urban visceral leishmaniasis in Brazil. Emerg. Infect. Dis..

[28] Casanova C., Andrighetti M.T., Sampaio S.M., Marcoris M.L., Colla-Jacques F.E., Prado A.P. (2013). Larval breeding sites of *Lutzomyia longipalpis* (Diptera: Psychodidae) in visceral leishmaniasis endemic urban areas in Southeastern Brazil. PLOS Negl. Trop. Dis..

[29] Nunes C.M., Pires M.M., Silva K.M., Assis F.D., Gonçalves Filho J., Perri S.H. (2010). Relationship between dog culling and incidence of human visceral leishmaniasis in an endemic area. Vet. Parasitol..

[30] Coura-Vital W., Marques M.J., Veloso V.M., Roatt B.M., Aguiar-Soares R.D., Reis L.E., Braga S.L., Morais M.H., Reis A.B., Carneiro M. (2011). Prevalence and factors associated with *Leishmania infantum* infection of dogs from an urban area of Brazil as identified by molecular methods. PLOS Negl. Trop. Dis..

[31] Mohebali M. (2013). Visceral Leishmaniasis in Iran: Review of the Epidemiological and Clinical Features. Iran. J. Parasitol..

[32] Parvizi P., Alaeenovin E., Mohammadi S., Baghban N. (2013). Occurrence of low density of *Leishmania infantum* in sandflies from a new focus of visceral leishmaniasis in northwest of Iran. J. Vector Borne Dis..

[33] Cassini R., Signorini M., Frangipane di Regalbono A., Natale A., Montarsi F., Zanaica M., Brichese M., Simonato G., Borgato S., Babiker A., Pietrobelli M. (2013). Preliminary study of the effects of preventive measures on the prevalence of Canine Leishmaniosis in a recently established focus in northern Italy. Ital. Vet..

[34] Carvalho G.M., Brasil R.P., Saraiva L., Quaresma P.F., Botelho H.A., Ramos M.C.N.F., Zenóbio A.P.A.L., Meira P.C.L.S., Sanguinette C.C., Andrade Filho J.D. (2012). Hourly activity and natural infection of sandflies (Diptera: Psychodidae) captured from the Aphotic Zone of a Cave, Minas Gerais State, Brazil. PLoS One.

[35] Romero G.A., Boelaert M. (2010). Control of visceral leishmaniasis in Latin America—A systematic review. PLOS Negl. Trop. Dis..

[36] Warburg A., Faiman R. (2011). Research priorities for the control of phlebotomine sand flies. J. Vector Ecol..

